# A systematic review of blunt abdominal aortic injury and analysis of predictors of death

**DOI:** 10.17305/bb.2023.9831

**Published:** 2024-06-01

**Authors:** Mingxuan Li, Chaonan Wang, Haixia Tu, Haitao Zhu, Zhen Guo, Lianrui Guo

**Affiliations:** 1 Department of Vascular Surgery, Beijing Fengtai You’anmen Hospital, Beijing, China; 2 Department of Vascular Surgery, Peking Union Medical College Hospital, Beijing, China; 3 Department of Vascular Surgery, Xuanwu Hospital Capital Medical University, Beijing, China

**Keywords:** Blunt abdominal aortic injury (BAAI), blunt trauma, abdominal injury, mortality, trauma

## Abstract

At present, research on blunt abdominal aortic injury (BAAI) is limited, with the majority being case reports. Consequently, there is a significant knowledge gap concerning this condition. To address this, we conducted a systematic review by extensively searching major databases. We included all literature that provided individual (non-identifiable) data on BAAI patients, irrespective of the study design. Furthermore, we undertook regression analyses to identify predictors of death after BAAI. The search yielded 2099 results, leading to the inclusion of 102 case reports and one conference abstract. Using the Joanna Briggs Institute (JBI) checklist for assessment, all studies were deemed of medium to high quality. In total, 133 patients were included, with a median age of 34 years, and 73.7% being male. The predominant clinical manifestation was pain, reported in 65.6% of patients. The most frequently observed aortic lesion severity was grade A (intimal tear or intramural hematoma) at 46.9%, and the most common lesion location was zone III (infrarenal aorta) in 88.3% of cases. The overall mortality after BAAI was 15.3%. Multivariate regression analyses revealed the following predictors of death after BAAI: lower limb ischemia (relative risk [RR] ═ 7.137, 95% confidence interval [CI] 1.154–44.161), cardiopulmonary arrest (RR ═ 10.250, 95% CI 1.452–72.344), and injuries to body parts other than the abdomen and lumbar spine (RR ═ 2.593, 95% CI 1.189–5.655). In conclusion, this review provides a detailed quantitative summary of BAAI’s clinical manifestations, diagnosis, treatment, and prognosis, emphasizing its high mortality rate and identifying three critical variables as predictors of death.

## Introduction

Blunt abdominal aortic injury (BAAI) is believed to be caused by an injury to the aorta due to both direct and indirect blunt biomechanical forces exerted on the abdominal aorta (AA). The AA is tethered between the spinal column and the peritoneum, along with the abdominal viscera [1]. BAAI is rare in both adults and children [2]. According to previous studies, it accounts for only 4%–6% of total aortic injuries and less than 1% of all blunt traumas [3–5]. Despite its rarity, BAAI is lethal. Previous research has highlighted a high but variable mortality rate, with figures ranging from 17% to 92% [5–8]. In comparison to blunt thoracic aortic injury (BTAI), BAAI is significantly less common. This is believed to result partly from the relatively fixed position of the entire AA, in contrast to the thoracic aorta, which is fixed only at the ligamentum arteriosum and the base of the heart [9].

Shalhub et al. [1] proposed a classification of aortic lesion locations into three types to facilitate surgical exposure and repair. This classification method has been favored by many researchers [10, 11]. On the other hand, when dealing with BTAI or nonspecific blunt aortic injury (BAI), various grading criteria have been developed to assess the severity of aortic lesions based on the pathological changes in the injured aorta [12–15]. Despite these advancements, limited research has explored the impact of aortic lesion location and severity on the prognosis of BAAI patients. The therapeutic approaches for BAAI mainly consisted of surgical intervention and conservative observation. Kondo et al. [16] found no statistically significant difference in 24-h mortality or overall hospital mortality rates among the different treatment modalities. However, Sheehan et al. [17] reported that patients who underwent aortic surgery exhibited a statistically lower mortality rate than those who did not. There is limited research in this area.

The limited existing studies have not extensively addressed the core aspects of BAAI, leaving significant gaps in the understanding of this dangerous traumatic condition. To address these gaps, we conducted this systematic review. This review was registered in the International Prospective Register of Systematic Reviews (PROSPERO) (CRD42023408842) and aligns with the guidelines of the Preferred Reporting Items for Systematic Reviews and Meta-Analyses (PRISMA) framework [18].

## Materials and methods

### Search strategy

**Figure S1.**
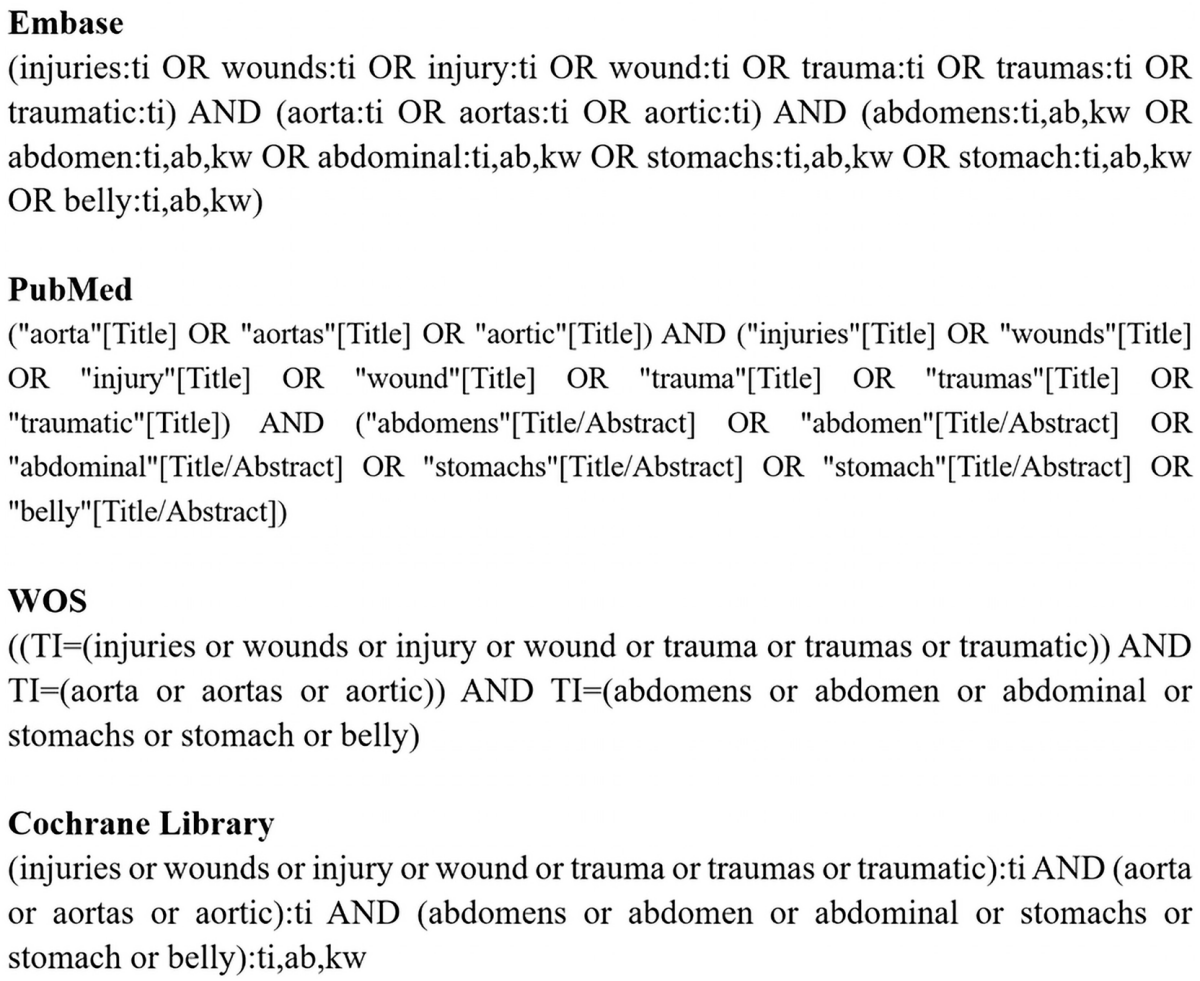
**Overview of search terms used in the literature search across different databases.** WOS: Web of Science.

A systematic search was conducted in the Excerpta Medica Database, PubMed, Web of Science (WOS), and Cochrane Library on December 23, 2022. All terms synonymous with or similar to “abdominal aortic injury (AAI)” were searched to ensure no relevant studies were overlooked. The detailed search strategy can be found in Figure S1.

### Study selection

The AA was defined as the section of the aorta extending from the diaphragm to where it bifurcates into the right and left common iliac arteries. The study subjects were human individuals diagnosed with BAAI, resulting from blunt or non-piercing external forces. All brief details from the identified articles were imported into Endnote X9 to facilitate the removal of duplicates and to conduct an initial brief review. Subsequently, the full texts of potentially available articles that passed the preliminary screening were downloaded for a more thorough review to identify those articles that were included in the study.

Initially, all articles pertaining to AAI that were retrieved from the primary search were considered. In further search, any literature providing individual data on BAAI patients, regardless of the study design, was eligible for inclusion, even if there were missing values for certain variables. The exclusion criteria were as follows: (1) subjects with concomitant thoracic aortic injury; (2) subjects with pre-existing dilative aortic conditions (such as aortic aneurysm) or aortic injury; (3) potential duplicate subjects; and (4) articles which were published in languages other than English. If a particular article reported on multiple BAAI cases and only some met the selection criteria, only those specific cases were included in this review. Two authors, ML and CW, independently performed the data selection process. Discrepancies between their selections were addressed and resolved through consensus.

### Data extraction

An extensive set of variables related to BAAIs was established for detailed data extraction. The definitions and descriptions of each variable can be found in Table S1. Specifically, “death after BAAI” was defined as any death resulting from the initial traumatic event, excluding other causes such as malignant cancer. Aortic lesion locations were categorized into three zones based on Shalhub et al.’s methodology [1]: zone I corresponding to the zone extending from the diaphragmatic hiatus to the superior mesenteric artery (SMA); zone II corresponding to the zone encompassing the SMA and the renal arteries; and zone III corresponding to the zone spanning from the infrarenal aorta to the aortic bifurcation. Furthermore, the severity of aortic injuries was classified into five grades building on the basis of Rabin et al.’s method [15]: grade A corresponding to intimal tear or intramural hematoma; grade B corresponding to small pseudoaneurysm (less than 50% circumference); grade C corresponding to large pseudoaneurysm (more than 50% circumference); grade D corresponding to intraluminal truncation; and grade E corresponding to rupture. Data extraction was performed by two independent authors, HT and HZ. If a variable’s value or classification was not explicitly reported in an included article based on our definitions, these authors would independently interpret the original text to determine it. Any queries and discrepancies in interpretation were addressed, and consensus was achieved through further discussion.

### Quality assessment

The methodological quality of the original studies was evaluated using the Joanna Briggs Institute (JBI) checklist for case reports [19]. This assessment tool includes eight items, which evaluate whether the descriptions of the patient’s clinical characteristics, diagnosis, treatment, outcomes, and other relevant aspects in each report are comprehensive and clear. Each item earns a score of 1 point for responses “yes” or “not applicable” and 0 points for responses “no” or “not clear”. In instances where a single study reported on multiple cases separately, each case was evaluated separately, and the overall score of the study was determined by the lowest score among its individual cases.

Based on their scores, all included studies were classified in terms of methodological quality as “low” (0–3 points), “medium” (4–6 points), or “high” (7–8 points). Three independent authors (ML, ZG, and LG) each carried out the quality assessments for all included articles. In cases of differing opinions, the lower score was taken into consideration.

### Ethical statement

Given that this review exclusively utilizes previously published literature and does not contain any personally identifiable information, neither ethical approval nor consent to participate was required, which is in line with prevailing local regulations and policies. Furthermore, our study adhered to the principles outlined in the Declaration of Helsinki (as revised in 2013).

### Statistical analysis

Continuous variables were expressed as either “mean ± standard deviation” or “median with interquartile range (IQR).” Categorical variables were presented as “number with percentage”. Statistical analyses were conducted using the Stata version 16.0 software (StataCorp., College Station, TX, USA). All hypothesis tests were two-sided, and a *P* value of < 0.05 was considered statistically significant. The Mann–Whitney *U* test [20] was employed to compare age distributions of BAAI patients across sexes. The constituent ratios for all samples in categorical variables were also calculated separately. The details of patients who died after BAAI were also described.

For assessing potential predictors, the binary “death after BAAI” outcome variable was set. All variables, excluding those who were deemed unrelated like “diagnostic method,” were individually incorporated into a binomial family generalized linear model (GLM) (link: log; standard error type: default; and optimization method: maximum likelihood estimation) for univariate regression analyses, with relative risk (RR) and 95% confidence interval (CI) as metrics [21]. To achieve a probable positive result and retain maximum detailed categorization, we tried multiple combinations of core treatments, incorporating each set of derived variables into the model individually. An adjusted multivariate regression analysis was initially attempted using the above GLM for variables with *P* < 0.20, but convergence was not achieved and no results were derived. Subsequently, a robust Poisson family GLM (link: log; standard error type: robust; and optimization method: maximum likelihood estimation) was used for multivariate regression analysis of these variables. Only variables with a *P* value < 0.05 and an absolute RR value < 1000 were accepted and considered as predictors. To assess the final adopted regression model’s fitting ability, we recorded the values of the Akaike information criterion (AIC) [22] and log pseudolikelihood [23]. Furthermore, subgroup analyses were conducted using Fisher’s exact test [24] on patients with the calculated predictors to explore the influence of different treatment modalities on mortality within these specific patients. All statistical analyses were conducted by ML.

## Results

### Characteristics of studies and patients

**Figure S2.**
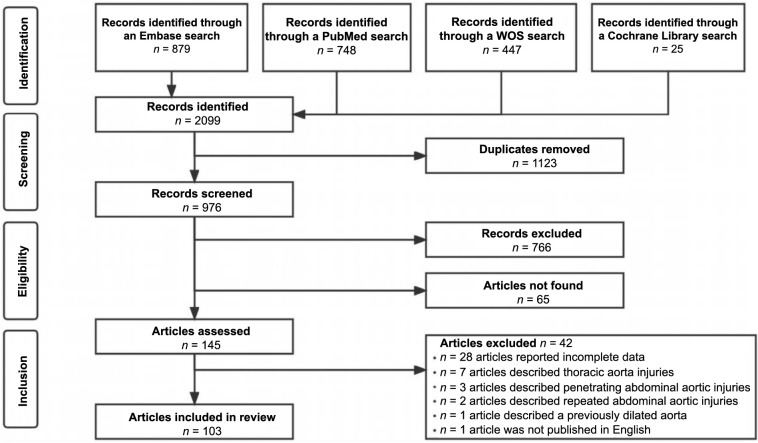
**PRISMA flowchart, showcasing the step-by-step process of study identification and inclusion for our systematic review.** PRISMA: Preferred Reporting Items for Systematic Reviews and Meta-Analyses; WOS: Web of Science.

Our systematic search initially identified 2099 articles. After removing 1123 duplicates, we screened the titles and abstracts of the remaining studies, excluding additional 766 articles. Subsequently, full-text assessment led to the final inclusion of 103 articles [2, 3, 25–124] (Figure S2).

**Figure S3.**
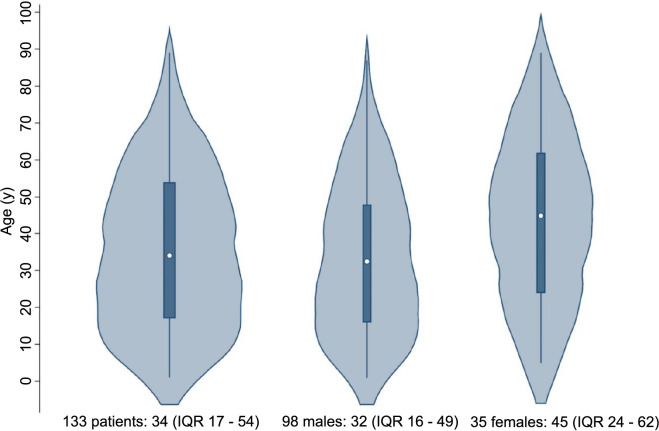
**Age distribution across sexes.** IQR: Interquartile range.

These articles comprised 102 case reports and one conference abstract. Among them, 14 articles (13.6%) reported on multiple cases, ranging from two to seven cases. In terms of quality assessment, 91 studies were deemed high-quality studies (88.3%), 12 studies were deemed medium-quality ones (11.6%), with none classified as low-quality studies. These articles were published from 1961 to 2021, with 83 (80.6%) published after 1990. They originated from 25 countries, with the USA contributing the most (48, 46.6%). One case involved an injury abroad with subsequent treatment in the USA later and was reported by American doctors [31]. We assumed that the rest of the patients were injured and, if treated, received their care in their respective reporting countries. The authors of one article published in 2018 were from Serbia and Montenegro, respectively [95], and the reporting country of this article was considered to be one rather than two. In total, 133 BAAI patients were included, with a median age of 34 years (IQR 17–54), and an age range of 1–89 years. The detailed characteristics of the included studies and patients can be found in Table S2. Among the patients, 35 were females (26.3%), who were generally older than males (*P* ═ 0.012) (Figure S3).

### Clinical manifestations, diagnoses, treatments, and outcomes

**Figure 1.**
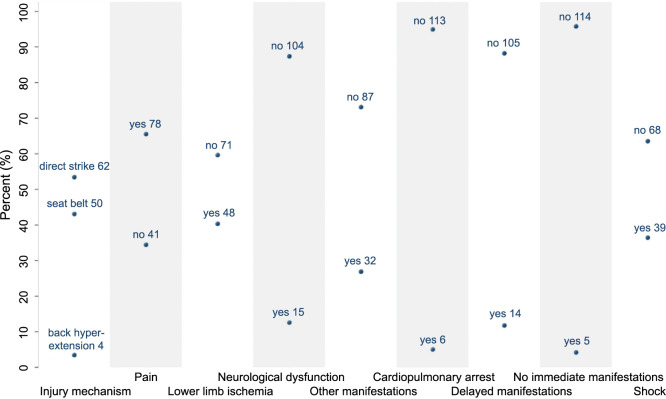
**The distribution of patients across each of the clinical manifestation variable.** The numbers of patients are labeled within the figure.

The predominant cause of BAAI in patients was “direct strike (non-seat belt),” such as collisions, falls, gas shocks, and more, accounting for 53.4% of cases. The various clinical presentations were classified into four categories, with pain emerging as the most common symptom (65.5%). Notably, 5.0% of patients presented with cardiopulmonary arrest and 36.4% with shock. Using the first day after trauma as a reference point, 11.8% of patients experienced delayed manifestations, whereas 4.2% showed no acute manifestations. The distribution of patients across various clinical manifestation variables is shown in Figure 1.

**Figure 2.**
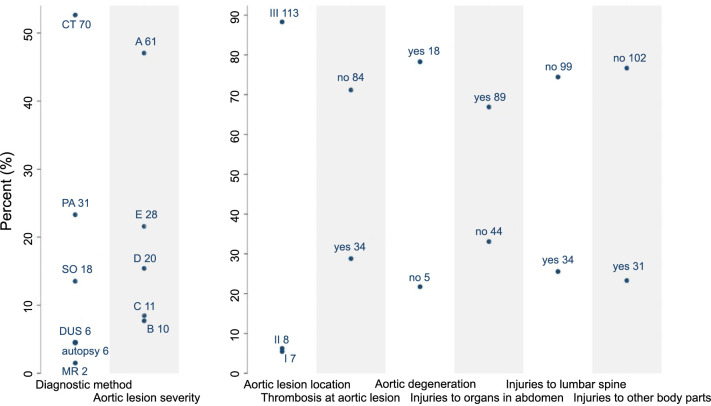
**The distribution of patients across each of the diagnostic variable.** The numbers of patients are labeled within the figure. CT: Computed tomography; PA: Percutaneous aortogram; SO: Surgical operation; DUS: Doppler ultrasound; MR: Magnetic resonance.

Computed tomography (CT) was the most commonly used diagnostic tool, utilized in 52.6% of patients. Three patients were diagnosed with BAAI after substantial delays of seven months, eight years, and nine years after trauma, respectively, due to different reasons [41, 43, 55]. Using the established classification systems, 88.3% of patients had an aortic lesion located in zone III, with 47.0% being of grade A severity. Thrombosis at the aortic lesion site was reported in 28.8% of cases, and 78.3% of cases were reported to have aortic degeneration. In addition, 66.9% of patients had concurrent injuries to other abdominal organs, 25.6% to the lumbar spine, and 23.3% to other body parts. Concomitant injuries to as many as seven organs or tissues after trauma were reported in one patient [99]. Among all abdominal organ injuries, the gastrointestinal tract was most frequently affected, accounting for 49.6% of all BAAI patients. The distribution of diagnostic variables is depicted in Figure 2.

**Figure 3.**
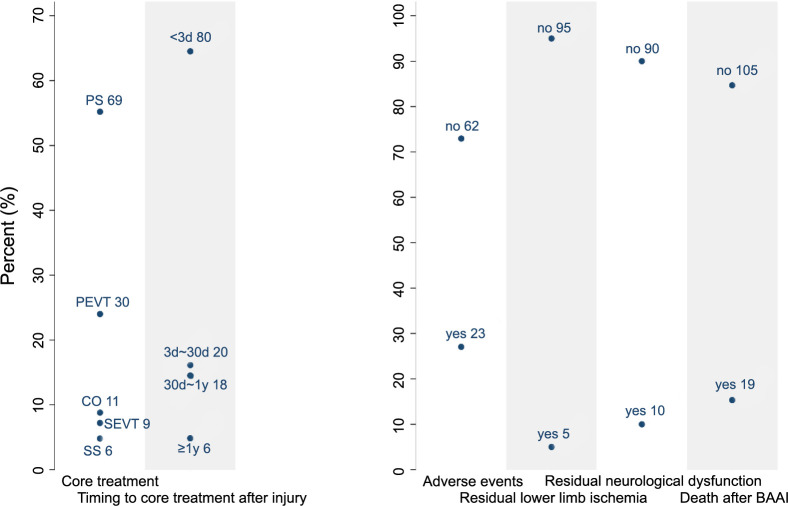
**The distribution of patients across each of the treatment modality and outcome variable.** The numbers of patients are labeled within the figure. PS: Primary surgery; PEVT: Primary endovascular therapy; CO: Conservative observation; SEVT: Secondary endovascular therapy; SS: Secondary surgery; BAAI: Blunt abdominal aortic injury.

The most common treatment modality for BAAI patients was primary open surgery (OS), accounting for 55.2% of cases, followed by primary endovascular therapy (EVT) at 24.0%, with other modalities making up the remainder. One patient initially underwent percutaneous aortic stent implantation after an unsuccessful conservative observation. However, during the procedure, it was discovered that the stent had migrated to the suprarenal aorta. Consequently, the patient underwent an open aorto-aortic bypass and made a successful recovery [88]. This patient was subsequently categorized as having undergone a secondary OS as the core treatment modality. Among the 114 patients who underwent surgical treatment, the decision of their treatment approach (OS or percutaneous EVT) was not statistically associated with the presence of neither injuries to abdominal organs, lumbar spine, or other body parts (*P* ═ 0.144; *P* ═ 0.240; and *P* ═ 0.486, respectively). Similarly, the incidence of gastrointestinal injuries, the most common abdominal trauma, did not significantly sway the choice between OS and EVT (*P* > 0.999). Of the 114 patients who underwent surgery, 60 patients had gastrointestinal injuries, and 29 of them underwent OS rather than EVT (*P* > 0.999). Regarding the timing of core treatments after injury, it was found that most patients (64.5%) received treatment rapidly, within three days after injury. One patient underwent immediate OS upon admission [43], while another patient received treatment in the ninth year after the injury due to a missed diagnosis [44]. The longest follow-up duration reported was ten years [102]. Post-treatment, 27.1% of patients experienced various non-fatal adverse events, with acute renal failure being the most common at 47.8%. Some patients reported up to four distinct adverse events following OS, excluding instances of multiple organ dysfunction syndrome [43, 45]. Residual chronic lower limb ischemia and neurological dysfunction were observed as post-treatment sequelae in 5.0% and 10.0% of patients, respectively. A total of 19 patients (15.3%) died after BAAI. They were classified based on the time of death (Table 1). The distribution of treatment modality and outcome variables are shown in Figure 3. Among the 53 cases with available data who had gastrointestinal injury and underwent surgical procedures, three died after BAAI in the OS group and none in the EVT group, which did not suggest a statistically significant difference (*P* ═ 0.543).

**Table 1:** Data from the 19 patients who died following a BAAI

**Death occurence**	***n* (%)**	**Reference**	**Age (years)**	**Sex**	**Injury mechanism**	**Clinical manifestations**	**Aortic lesion characteristics**	**Other injuries**	**Core treatment and its timing**	**Time to death after injury**	**Immediate cause of death**	**Adverse events**
Before diagnosis	5 (26.3)	Coimbra et al. [81]	70	M	DS	CA, S	Z III, G E, T, AD	A, LP, O	–	<1 day	Hemorrhage	–
		Coimbra et al. [81]	2	M	DS	CA, S	G E	A, LP, O	–	<1 day	Hemorrhage	–
		Coimbra et al. [81]	19	M	DS	CA, S	G E	A, O	–	<1 day	Hemorrhage	–
		David et al. [99]	54	F	DS	P, LLI	Z II, G A, T	A, O	–	2 days	Respiratory failure	–
		Desai et al. [113]	78	F	DS	CA, S	Z II, G A, AD	No	–	<1 day	Hemorrhage	–
After diagnosis and before initial treatment	1 (5.3)	Harkin et al. [32]	7	M	DS	P, LLI, S	Z III, G E	No	–	<1 day	Hemorrhage	–
During operation	2 (10.5)	Hewitt et al. [36]	65	M	SB	LLI, OM	Z III, G D, T, AD	A	POS, <3d	<1 day	N/A	N/A
		Gordon et al. [114]	46	F	DS	CA, S	Z II, G E	LP	POS, <3d	<1 day	Hemorrhage	Spinal cord injury
After initial treatment	10 (52.6)	Reisman et al. [38]	55	F	DS	P, LLI, ND, OM	Z III, G E, T, AD	A, O	POS, <3d	2 months	MODS	MODS
		Bergqvist et al. [43]	66	F	SB	P, LLI	Z III, G E, T, AD	No	POS, <3d	9 days	Septic shock	MODS, poor wound healing
		Sadaghianloo et al. [2]	15	M	DS	N/A	Z III, G B	A	CO, <3d	<1 day	Hemorrhage	N/A
		Frydenburg et al. [53]	78	M	SB	LLI	G A, T	O	CO, <3d	<1 day	Respiratory and renal failure	Respiratory and renal failure
		Frydenburg et al. [53]	71	F	SB	LLI	Z III, G D	O	CO, <3d	2 days	Respiratory and renal failure	Respiratory and renal failure
		Michaels et al. [25]	60	F	SB	LLI, OM, S	Z III, G D	A	POS, <3d	4 days	N/A	N/A
		Nucifora et al. [3]	36	M	DS	OM, S	Z III, G E	No	POS, <3d	<1 day	Hemorrhage	N/A
		Tracy et al. [63]	13	M	DS	OM, S	Z III, G E	O	POS, <3d	<1 day	Hemorrhage	No
		Macbeth et al. [90]	60	M	DS	P, LLI, ND	Z III, G D, T, AD	A	POS, 30d-1y	1.5 months	Septic shock	Septic shock, renal failure
		Warrian et al. [97]	36	F	SB	LLI, OM	Z III, G E, AD	O	POS, <3d	19 days	MODS	MODS
On missed diagnosis	1 (5.3)	Katsoulis et al. [58]	41	M	DS	P, LLI, ND	N/A	A, LP	–	9 days	Hemorrhage	No

BAAI: Blunt abdominal aortic injury; M: Male; F: Female; DS: Direct strike; SB: Seat belt; CA: Cardiopulmonary arrest; S: Shock; P: Pain; LLI: Lower limb ischemia; OM: Other manifestations; ND: Neurological dysfunction; N/A: Not available; Z: Zone; G: Grade; T: Thrombosis; AD: Aortic degeneration; A: Abdominal organs injuries; LP: Lumbar spine injuries; O: Other body parts injuries; POS: Primary open surgery; CO: Conservative observation; MODS: Multiple organ dysfunction syndrome.

### Death after blunt abdominal aortic injury

**Figure S4.**
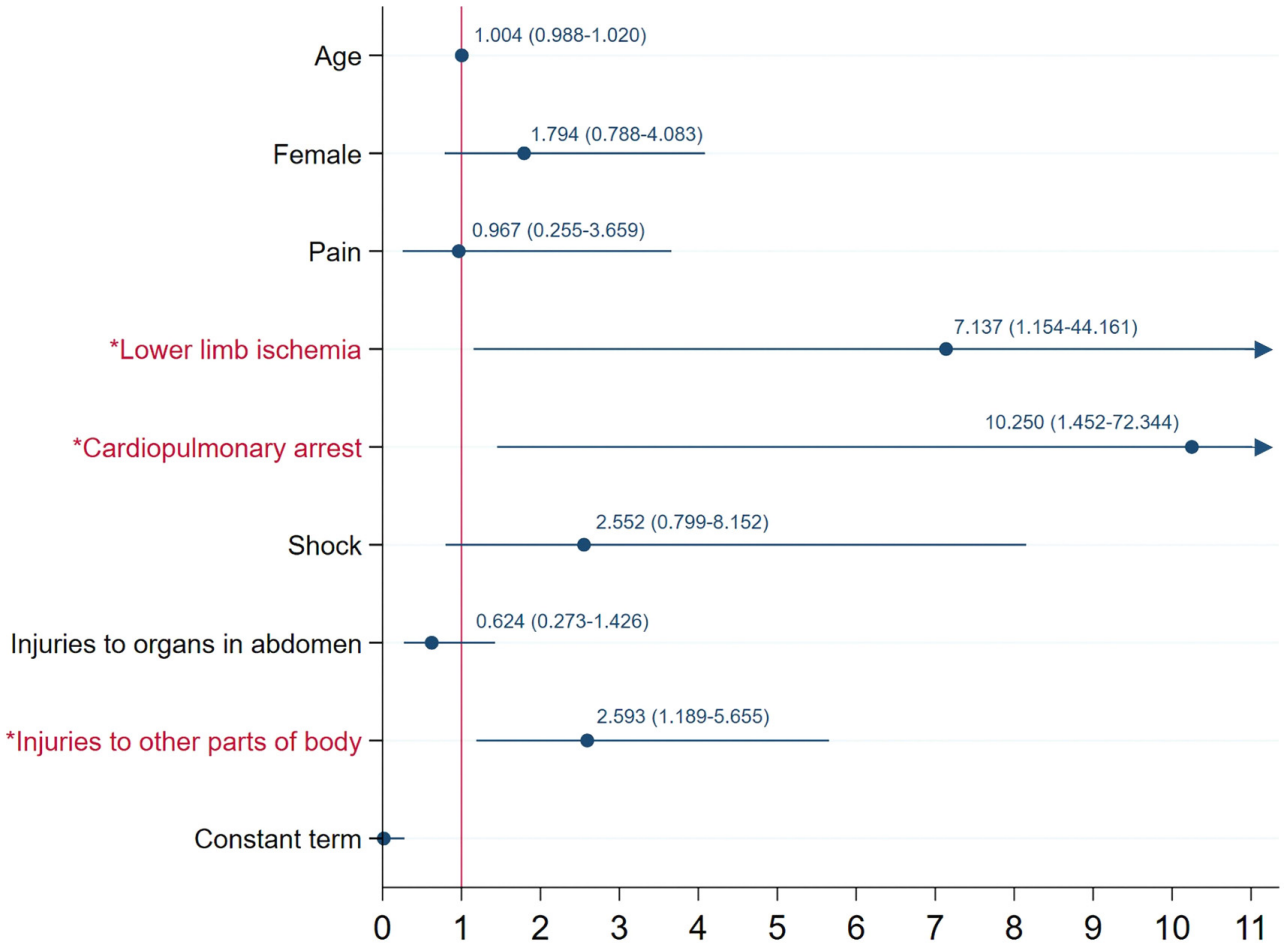
**Forest plot illustrating the relative risks (with 95% confidence intervals) of adopted predictors of death after BAAI.** ^*^Statistically significant variable (*P* < 0.05). BAAI: Blunt abdominal aortic injury.

Due to the failed convergence of the binomial regression model after the selections of variables through univariate analyses, a robust Poisson GLM was employed for analysis, which derived several variables with *P* < 0.05. However, the absolute RR values of some of these variables, including all the multi-categorical ones, were exaggeratedly exceeding 1000, with some even reaching trillions. To pinpoint variables that destabilized the model, we utilized multiple stepwise regression approaches, such as forward and backward regression. After excluding all multicategorical variables, a stable Poisson regression model emerged, encompassing 86 observations. The model had an AIC value of 1.018 and a log pseudolikelihood value of −34.776. We collected all the variables in this model and tried the binomial regression again, but the binomial regression still could not achieve convergence. Thus, we ultimately utilized the Poisson regression model, and three statistically significant predictors (or risk factors) of death after BAAI were identified, which were lower limb ischemia (RR ═ 7.137, 95% CI 1.154–44.161; *P* ═ 0.035), cardiopulmonary arrest (RR ═ 10.250, 95% CI 1.452–72.344; *P* ═ 0.020), and injuries to other body parts (RR ═ 2.593, 95% CI 1.189–5.655; *P* ═ 0.017). A detailed breakdown of the analysis is presented in Table 2. The adopted model results are visualized in a forest plot (Figure S4).

**Table 2:** Univariate and multivarite analysis exploring variables associated with death after BAAI

		**Univariate analysis**		**Multivariate analysis 1^†^**		**Multivariate analysis 2^‡^**	
**Variable**	**No. of obs**	**Relative risk (95% CI)**	***P* value**	**Relative risk (95% CI)**	***P* value**	**Relative risk (95% CI)**	***P* value**
Age (years)	124	1.018 (1.001 – 1.035)	**0.040**	1.054 (1.019 – 1.090)	**0.002**	1.004 (0.988 – 1.020)	0.598
Female	124	2.182 (0.966 – 4.929)	**0.061**	0.494 (0.098 – 2.499)	0.394	1.794 (0.788 – 4.083)	0.164
Injury mechanism	104						
Direct strike		Reference					
Seat belt		0.851 (0.335 – 2.166)	0.735				
Back hyperextension		1	–				
Pain	110	0.264 (0.108 – 0.648)	**0.004**	0.263 (0.100 – 0.689)	**0.007**	0.967 (0.255 – 3.659)	0.960
Lower limb ischemia	110	2.106 (0.883 – 5.023)	**0.093**	8.170 (0.738 – 90.486)	0.087	7.137 (1.154 – 44.161)	**0.035**
Neurological dysfunction	110	1.267 (0.416 – 3.857)	0.677				
Other manifestations	110	1.397 (0.577 – 3.379)	0.459				
Cardiopulmonary arrest	110	6.667 (3.580 – 12.415)	**<0.001**	2.70e^+07^ (1.62e^+06^ ∼4.50e^+08^)	**<0.001**	10.250 (1.452 – 72.344)	**0.020**
Delayed manifestations	110	0.439 (0.064 – 3.030)	0.403				
No immediate manifestations	106	1	–				
Shock	99	2.207 (0.976 – 4.990)	**0.057**	2.715 (0.603 – 12.224)	0.193	2.552 (0.799 – 8.152)	0.114
Aortic lesion location	113						
Zone I		1	–	9.28e^-08^ (1.86e^-08^ ∼4.64e^-07^)	**<0.001**		
Zone II		3.281 (1.159 – 9.293)	**0.025**	2.82e^-07^ (5.07e^-08^ ∼1.57e^-06^)	**<0.001**		
Zone III		Reference		Reference			
Aortic lesion severity	111						
Grade A		Reference		Reference			
Grade B		4.143 (0.429 – 40.050)	0.219	5.97e^+05^ (9.29e^+04^ ∼3.84e^+06^)	**<0.001**		
Grade C		1	–	2.93e^+05^ (2.59e^+04^ ∼3.33e^+06^)	**<0.001**		
Grade D		6.444 (1.284 – 32.334)	**0.024**	8.45e^+11^ (1.09e^+11^ ∼6.58e^+12^)	**<0.001**		
Grade E		11.393 (2.706 – 47.968)	**0.001**	2.06e^+12^ (2.99e^+11^ ∼1.42e^+13^)	**<0.001**		
Thrombosis at aortic lesion	110	1.750 (0.659 – 4.648)	0.261				
Aortic degeneration	23	0.972 (0.287 – 3.292)	0.964				
Injuries to abdominal organs	124	0.569 (0.251 – 1.292)	**0.178**	0.909 (0.399 – 2.071)	0.821	0.624 (0.273 – 1.426)	0.263
Injuries to lumbar spine	124	0.914 (0.330 – 2.534)	0.863				
Injuries to other body parts	124	2.700 (1.209 – 6.031)	**0.017**	4.775 (0.387 – 58.855)	0.222	2.593 (1.189 – 5.655)	**0.017**
Core treatment	102						
Primary operation		0.363 (0.115 – 1.142)	**0.083**	2.882 (0.519 – 15.994)	0.226		
Secondary operation		1	**–**	3.12e^+04^ (7.06e^+03^ ∼1.38e^+05^)	**<0.001**		
Conservative observation		Reference		Reference			
Timing to core treatment after injury	91						
< 3d		Reference					
3d – 30d		1	–				
30d – 1y		0.363 (0.051 – 2.603)	0.313				
≥ 1y		1	–				
Adverse events	23	1	–				
Residual lower limb ischemia	95	1	–				
Residual neurological dysfunction	10	1	–				

^†^All variables with a *P* value < 0.20 from the univariate analysis (whose *P* values are bolded in the table under the univariate analysis) were included in this model. ^‡^All variables with a *P* value < 0.20 from the univariate analysis and an RR value < 1000 from multivariate analysis 1 were included in this model. The bolded *P* values under both multivariate analysis models represent statistically significant values (*P* < 0.05). BAAI: Blunt abdominal aortic injury; No. of obs: Number of observations; CI: Confidence interval.

Upon conducting subgroup analyses, it was found that BAAI patients with either lower limb ischemia or injuries to other body parts had lower mortality rates with both primary and secondary operations compared to those who received conservative observation (*P* ═ 0.020 and *P* ═ 0.035, respectively). Conversely, for patients with cardiopulmonary arrest, mortality rates did not differ significantly across different treatment modalities (*P* > 0.999). Detailed findings are shown in Table S3.

## Discussion

### Significance of this study

This review elucidated the multifaceted characteristics of BAAIs and identified risk factors associated with death. Such insights could help clinicians to more effectively recognize this rare yet fatal traumatic condition and to provide timely and accurate treatment for high-risk patients.

### Characteristics of blunt abdominal aortic injuries

Most of the studies regarding BAAI are case reports, underscoring the rarity of the condition. Among the 133 included BAAI patients, the median age for males was significantly lower than that for females (32 years vs 45 years; *P* ═ 0.012). This discrepancy might be attributed to the inclination of men in adolescence or youth toward high-intensity activities, such as driving motor vehicles, playing football, and boxing.

A direct blunt external force of sufficient magnitude can cause BAAI, and this type of trauma accounted for the majority (53.4%) of causes in this review. However, a significant portion of patients (43.1%) did not experience a direct crash, instead, they were restricted by the seat belt and faced injuries during sudden deceleration, thereby developing BAAI. Such injuries are believed to result from the combined compressive effect on the AA by the anteriorly located abdominal viscera and the posteriorly located lumbar spine [96, 125]. Meanwhile, we also found four patients (3.4%) who developed BAAI without suffering any direct external force applied to the abdomen, attributing their injuries to back hyperextension which caused excessive AA traction [79, 80, 85, 97]. Additionally, regardless of the injury type, damage to the lumbar spine may promote the development of BAAI due to the increased local force on the AA [46, 47, 79]. The presented review showed that the clinical manifestations of BAAI patients are diverse. Severe cases may present with shock or cardiopulmonary arrest, but pain, lower limb ischemia, and neurological dysfunction were representative. Among them, the proportion of patients with reported pain in the abdomen, chest, back, or lumbar flakes reached 65.5%, suggesting that pain may be the most common clinical symptom of BAAI patients. In addition to pain and lower limb ischemia due to reduced blood flow, 12.6% of patients exhibited neurological dysfunctions at different degrees, including paraplegia, hypoesthesia, asynodia, and so on. These complications may arise from the direct injury to the lumbosacral spinal cord or plexus, or the damage of the Adamkiewicz artery, which originates from the AA and supplies blood directly to the lumbosacral spinal cord [126].

The majority (52.6%) of BAAIs were diagnosed using CT. This underscores the significance of CT as the most important imaging modality for the diagnosis of BAAI, which is also in line with the findings of many researchers [10, 127]. Up to 88.3% of patients exhibited aortic lesions in zone III, spanning from the infrarenal aorta to the aortic bifurcation. In contrast, only 15 cases presented with injuries located in zone I (above the SMA) and zone II (encompassing SMA and renal arteries). This could be attributed to the infrarenal AA being less protected compared to the suprarenal AA [128]. However, following the regression analyses, there was not enough evidence to prove that different injury locations affected the mortality rate of BAAI. Since the severity grading criteria for aortic lesions are not uniform [10, 15, 129], after comparing them, we adopted Rabin et al.’s [15] method specific to BAAI. Yet, under this grading, intimal tears, categorized as the mildest grade (grade A), left large aortic dissections (AD), with or without thrombosis leading to luminal obstructions, unaccounted for. Consequently, we introduced this severe form of AD as grade D, positioned just below grade E (rupture) in terms of severity, based on this standard. Even after this inclusion, the proportion of BAAI patients of the mildest grade (grade A) remained the highest (47.0%), indicating that most BAAIs result in mild AA damage. Aortic degenerative pathology was present in 78.3% of patients, while 25.6% reported lumbar spine fractures, with relevant reports. These two conditions, based on the compression pathogenic mechanism theory of the AA, may increase the susceptibility of trauma patients to BAAI, a hypothesis in line with the ideas of several studies [18, 128].

The prevailing treatment preference for BAAI among most clinicians is primary OS, accounting for 55.2%. When considering primary operations, including primary EVT, this proportion even reaches 79.2%. These statistics indicate a limited application of conservative treatments in BAAI. However, it is evident that not all BAAI patients encounter identical risks. Shalhub et al. [1, 6] believed that the decision to operate and the choice of surgical modality should be chosen dependent on the location and severity of the aortic injury. Moreover, findings from this review suggest that the choice of surgical approach by clinicians is not influenced by the presence of injuries to other organs or tissues, especially abdominal ones. We believe that this tendency can be partly attributed to the established practices and preferences of individual medical centers and clinicians.

Generally, the underlying causes of some unwanted or unexpected events in BAAIs could not be solely explained as traumatic or iatrogenic. Consequently, we collectively referred to these negative events, which occurred after treatment (including conservative observation), as “adverse events” (excluding death). We observed that the incidence of these adverse events stood at 27.1%, aligning closely with findings from a prior study [17]. Excluding deaths unrelated to BAAI (such as those due to malignancies years later), the mortality after BAAI in this presented review was 15.3% (*n* ═ 19). The immediate cause of death among these patients was mostly hemorrhage resulting from the AA injury, even in cases that had received treatment.

### Predictors of death after blunt abdominal aortic injury

The analysis results indicate an elevated risk of death after BAAI in patients presenting with lower limb ischemia, cardiopulmonary arrest, or injuries to other body parts. We believe that only severe AA injuries, such as those of grade D/E, are sufficient to cause lower extremity ischemic symptoms. Thus, the presence of lower limb ischemia can serve as a direct indicator of the ischemic severity resulting from aortic injury. Acute limb ischemia has long been associated with many serious consequences, including death, as substantiated by previous studies [130, 131]. The preliminary multivariable model did suggest that the severity of aortic disease (a multiple categorical variable) had a direct impact on mortality (*P* < 0.001), but it was not adopted due to its unusual RR values (over 1000). On the other hand, both cardiopulmonary arrest and injuries to other body parts signify the severity of trauma to the whole body, not limited to the AA. In essence, concurrent injuries could potentially aggravate the impact of an AA injury on the body or even lead to death. It is very difficult to pinpoint the specific initial cause of death in a patient with multiple injuries. Several variables were also excluded from our model due to their excessively high RR values which exceeded 1000. For instance, the mortality rates associated with zone I and II injuries, which are more difficult to access or control through OS, were higher compared to those of zone III injuries. Moreover, secondary EVT following unsuccessful conservative observation was associated with increased mortality compared to consistent conservative treatment. These findings, despite not being statistically valuable, should not be easily negated given their theoretical plausibility. The unusual RR values are likely a result of the small sample size. We anticipate future research with larger sample sizes to provide more clarity on these matters.

Subgroup analysis results suggest that for BAAI patients with either lower limb ischemia or injuries to other body parts, both primary and secondary operative treatments (including OS and EVT) can reduce the mortality risk compared to conservative observation (*P* < 0.05). This underscores the importance of surgical intervention for high-risk BAAI patients. However, for BAAI patients presenting with cardiopulmonary arrest, the differences in protective effects between the three treatment modalities were not evident. Given the small sample size (*n* ═ 2) for this subgroup, it is inconclusive to state that surgical interventions do not play a role in reducing the risk of death for such BAAI patients.

### Limitations

This review also has certain limitations. Firstly, due to the absence of uniform standards across the references, variable descriptions were occasionally vague, potentially affecting the accuracy of data extraction and subsequent analysis. Secondly, cases with favorable outcomes may be more readily reported and published, while those with less satisfactory outcomes might go unreported for various reasons. This could potentially lead to an underestimation of the true mortality rate, introducing publication bias.

## Conclusion

BAAI represents a lethal injury with diverse characteristics and a minimum mortality rate of 15.3%. Factors, such as lower limb ischemia, cardiopulmonary arrest, and injuries to body parts beyond the abdomen and lumbar spine increase the mortality risk associated with BAAI. Surgical intervention, whether through OS or EVT, can reduce the mortality in BAAI patients exhibiting lower limb ischemia or injuries to other body parts, even when used as a salvage measure following unsuccessful conservative observation.

## Supplemental data

**Table S1:** Necessary definitions and descriptions of variables

**Variable**	**Definition and description**
Injury mechanism	
Direct strike	Including motor vehicle crash (driver or passenger), fall, hit, airflow impact, or squeeze.
Seat belt	Seat belt was tied when trauma.
Back hyperextension	Back hyperextension without external forces.
Pain	Pain in abdomen, chest, back, or lumbar flanks.
Lower limb ischemia	Including pain, weakness, coldness, pulselessness, or pallor of lower extremity due to ischemia.
Neurological dysfunction	Including paraplegia, hypoesthesia, asynodia, incontinence, etc.
Other manifestations	Other manifestations due to trauma not covered by the previous two variables, including gastrointestinal symptoms, consciousness disorders, vertigo, headache, weakness, hyperhidrosis, dyspnea, etc.
Delayed manifestations	Manifestations that occurred more than 1d after trauma.
No immediate manifestations	No manifestations were observed within 1d following trauma.
Shock	Systolic blood pressure was < 90 mmHg.
Aortic lesion location	
Zone I	The zone from the diaphragmatic hiatus to the SMA.
Zone II	The zone encompassing the SMA and the renal arteries.
Zone III	The zone spanning from the infrarenal aorta to the aortic bifurcation.
Aortic lesion severity	
Grade A	Intimal tear or intramural hematoma.
Grade B	Small pseudoaneurysm (less than 50% circumference).
Grade C	Large pseudoaneurysm (more than 50% circumference).
Grade D	Intraluminal truncation.
Grade E	Rupture.
Aortic degeneration	Referred degenerative pathologies such as severe aortic calcification or atherosclerosis reported by imaging studies, pathological examination, or autopsy.
Injuries to organs in abdomen	Injuries to other vital organs or tissues in the abdomen such as the abdominal wall, pelvis, diaphragm, omentum, stomach, intestines and mesentery, liver, pancreas, spleen, kidney, etc.
Injuries to lumbar spine	All injuries to lumbar spine which were referred, including fracture or deformation.
Injuries to other body parts	Injuries to other body parts not covered by the previous two variables, including the head, thorax, thoracic spine, heart, lung, ribs, clavicles, or limbs.
Core treatment	There are five core treatment modalities. Both surgery and intervention were defined as ”operation”.
Primary surgery	The aorta was repaired, replaced, or bypassed under direct vision through incisional approach proactively after diagnosis of BAAI.
Secondary surgery	After the diagnosis of BAAI, the patient underwent initial conservative observation and then was switched to surgery passively.
Primary endovascular therapy	Endovascular repair of aorta with covered stent under the monitoring of aortography was performed through percutaneous puncture approach proactively after diagnosis of BAAI.
Secondary endovascular therapy	After the diagnosis of BAAI, the patient underwent initial conservative observation and then was switched to intervention passively.
Conservative observation	Conservative treatment was implemented throughout while monitoring for changes.
Adverse events	Refers to any unexpected adverse events other than death following core treatment, such as respiratory diseases, renal failure, heart diseases, infection, lower limb ischemia, neurological dysfunction, peptic ulcer, osteofascial compartment syndrome, poor incision healing, etc.
Death after BAAI	Death was primarily attributed to trauma, excluding other causes such as malignant cancer.

The table details an extensive set of variables related to BAAIs established for data extraction. Other variables included in the study were: Age, sex, cardiopulmonary arrest, diagnostic method, timing to core treatment after injury, residual lower limb ischemia, and residual neurological dysfunction. BAAI: Blunt abdominal aortic injury; SMA: Superior mesenteric artery.

**Table S2:** Basic characteristics of the included studies and patients

**Reference number**	**JBI score**	**Study type**	**Publication year**	**Country**	**Sample size**	**Age**	**Sex**
[25]	7	CR	2008	Japan	1	30	Male
[26]	8	CR	2006	Spain	1	18	Male
[27]	8	CR	2014	Brazil	1	5	Female
[28]	6	CR	2000	USA	1	21	Male
[29]	6	CR	1995	UK	3	15	Male
						16	Male
						21	Female
[30]	8	CR	2003	USA	1	6	Female
[31]	8	CR	1993	Dominica/USA	1	4	Male
[32]	8	CR	1999	UK	1	7	Male
[33]	6	CR	2014	USA	1	54	Male
[34]	8	CR	1969	USA	1	33	Female
[35]	7	CR	2011	USA	1	6	Female
[36]	8	CR	1970	USA	1	65	Male
[37]	8	CR	2007	Japan	1	66	Male
[38]	6	CR	1990	USA	2	17	Male
						55	Female
[39]	7	CR	2011	USA	1	2	Male
[40]	8	CR	2015	Israel	1	2	Male
[41]	8	CR	1983	Israel	1	39	Male
[42]	7	CR	2008	USA	1	10	Male
[43]	8	CR	1981	Sweden	2	66	Female
						33	Male
[44]	8	CR	1992	UK	1	19	Male
[45]	7	CR	1987	USA	1	52	Male
[46]	7	CR	2020	USA	1	14	Male
[47]	8	CR	2009	The Netherlands	1	18	Male
[48]	7	CR	2016	Japan	1	70	Female
[49]	7	CR	2021	New Zealand	1	12	Male
[50]	7	CR	2019	Taiwan, China	1	41	Male
[51]	8	CR	2018	Sweden	1	24	Female
[52]	8	CR	1993	USA	1	12	Male
[2]	6	CR	2014	France	3	15	Male
						7	Female
						4	Male
[53]	7	CR	1990	Australia	4	78	Male
						71	Female
						58	Female
						29	Female
[25]	7	CR	1996	USA	6	62	Male
						60	Female
						43	Female
						63	Male
						38	Male
						23	Male
[54]	7	CR	1997	USA	2	21	Female
						16	Male
[3]	8	CR	2008	Italy	1	36	Male
[55]	5	CR	1997	Spain	1	62	Female
[56]	7	CR	1989	USA	1	33	Female
[57]	7	CR	2003	USA	1	60	Male
[58]	6	CR	2006	UK	1	41	Male
[59]	8	CR	1990	UK	1	19	Male
[60]	8	CR	2004	USA	1	29	Male
[61]	7	CR	2006	USA	1	3	Male
[62]	8	CR	1997	Japan	1	67	Male
[63]	8	CR	1996	USA	1	13	Male
[64]	8	CR	2003	France	7	34	Male
						89	Female
						41	Male
						54	Male
						18	Male
						41	Male
						48	Male
[65]	8	CR	1997	France	3	34	Male
						89	Female
						41	Male
[66]	8	CR	2021	China	1	56	Male
[67]	8	CR	2021	Japan	1	78	Male
[68]	8	CR	2006	USA	1	56	Male
[69]	8	CR	2015	Greece	1	9	Male
[70]	8	CR	2009	USA	1	32	Male
[71]	7	CR	1998	France	3	34	Male
						89	Female
						41	Male
[72]	7	CR	2012	USA	1	21	Male
[73]	7	CR	2001	USA	1	21	Male
[74]	7	CR	2004	USA	1	54	Female
[75]	7	CR	2005	USA	1	26	Male
[76]	8	CR	2012	Japan	1	62	Male
[77]	8	CR	1991	Saudi Arabia	1	29	Male
[78]	8	CR	1975	USA	1	46	Male
[79]	8	CR	1997	Australia	1	21	Male
[80]	8	CR	2010	USA	1	16	Male
[81]	8	CR	1996	USA	3	70	Male
						2	Male
						19	Male
[82]	8	CR	1996	USA	1	34	Male
[83]	7	CR	2018	Sweden	2	52	Male
						57	Male
[84]	8	CR	2015	USA	1	53	Male
[85]	8	CR	2012	Poland	1	58	Male
[86]	8	CR	2007	USA	1	7	Male
[87]	8	CR	2005	Finland	1	61	Male
[88]	8	CR	2017	Spain	1	13	Male
[89]	8	CR	2015	USA	1	12	Male
[90]	8	CR	1982	USA	1	60	Male
[91]	7	CR	2000	Japan	1	38	Male
[92]	8	CR	1974	Greece	1	36	Male
[93]	8	CR	2012	Italy	1	66	Female
[94]	7	CR	2000	USA	1	28	Male
[95]	8	CR	2018	Serbia/Montenegro	1	18	Female
[96]	8	CR	1985	UK	1	54	Male
[97]	8	CR	1988	Canada	1	36	Female
[98]	7	CR	1969	UK	1	24	Male
[99]	7	CR	1970	USA	1	54	Female
[100]	8	CR	2005	USA	1	45	Female
[101]	8	CR	2018	South Korea	1	47	Male
[102]	7	CR	2011	Canada	1	15	Male
[103]	7	CR	1997	UK	1	17	Male
[104]	8	CR	1970	USA	1	45	Male
[105]	8	CR	2009	USA	1	1	Male
[106]	8	CR	2007	Israel	1	13	Male
[107]	6	CR	2007	USA	1	31	Male
[108]	7	CR	1971	USA	2	38	Male
						38	Female
[109]	8	CR	2013	Switzerland	1	11	Male
[110]	6	CR	1974	USA	2	32	Male
						28	Female
[111]	7	CR	2005	Morocco	1	5	Female
[112]	7	CR	1965	Nigeria	1	37	Female
[113]	8	CR	2008	USA	1	78	Female
[114]	8	CR	2007	USA	1	46	Female
[115]	7	CR	1988	USA	1	87	Male
[116]	8	CA	2014	USA	1	12	Male
[117]	7	CR	2014	Germany	1	54	Female
[118]	8	CR	2010	France	1	38	Male
[119]	8	CR	1961	UK	1	48	Male
[120]	6	CR	2019	Italy	1	47	Female
[121]	8	CR	2017	UK	1	42	Male
[122]	6	CR	1987	USA	1	17	Male
[123]	6	CR	2012	USA	1	35	Male
[124]	8	CR	2017	Serbia	1	51	Male

JBI: Joanna Briggs Institute; CR: Case report; CA: Conference abstract; USA: United States of America; UK: United Kingdom.

**Table S3:** Subgroup analysis evaluating the effect of core treatment modalities on death after BAAI

**Subgroup**	**Number of patients with or without death after BAAI^†^**			***P* value**
	**Primary operation**	**Secondary operation**	**Conservative observation**	
Lower limb ischemia +	6/29	0/7	2/0^‡^	**0.020**
Cardiopulmonary arrest +	1/0	0/0	0/1	>0.999
Injuries to other body parts +	3/17	0/5	2/0^‡^	**0.035**

^†^Expressed in: Number of patients who died after BAAI/number of patients who did not die after BAAI. ^‡^Mortality in this group differed significantly from the other two groups, respectively. Bolded *P* values represent statistically significant values (*P* < 0.05). BAAI: Blunt abdominal aortic injury.

## Data Availability

The original data is available from the corresponding author upon reasonable request.

## References

[1] Shalhub S, Starnes BW, Tran NT, Hatsukami TS, Lundgren RS, Davis CW (2012). Blunt abdominal aortic injury. J Vasc Surg.

[2] Sadaghianloo N, Jean-Baptiste E, Breaud J, Declemy S, Kurzenne JY, Hassen-Khodja R (2014). Blunt abdominal aortic trauma in paediatric patients. Injury.

[3] Nucifora G, Hysko F, Vasciaveo A (2008). Blunt traumatic abdominal aortic rupture: CT imaging. Emerg Radiol.

[4] Billy LJ, Amato JJ, Rich NM (1971). Aortic injuries in Vietnam. Surgery.

[5] de Mestral C, Dueck AD, Gomez D, Haas B, Nathens AB (2012). Associated injuries, management, and outcomes of blunt abdominal aortic injury. J Vasc Surg.

[6] Shalhub S, Starnes BW, Brenner ML, Biffl WL, Azizzadeh A, Inaba K (2014). Blunt abdominal aortic injury: a Western Trauma Association multicenter study. J Trauma Acute Care Surg.

[7] Lin CC, Liu KS, Chen HW, Huang YK, Chu JJ, Tsai FC (2016). Blunt aortic injury: risk factors and impact of surgical approaches. Surg Toda.

[8] Deree J, Shenvi E, Fortlage D, Stout P, Potenza B, Hoyt DB (2007). Patient factors and operating room resuscitation predict mortality in traumatic abdominal aortic injury: a 20-year analysis. J Vasc Surg.

[9] Randhawa MP Jr, Menzoian JO (1990). Seat belt aorta. Ann Vasc Surg.

[10] Prado E, Chamorro EM, Marín A, Fuentes CG, Zhou ZC (2022). CT features of blunt abdominal aortic injury: an infrequent but life-threatening event. Emerg Radiol.

[11] Tsai R, Raptis D, Raptis C, Mellnick VM (2018). Traumatic abdominal aortic injury: clinical considerations for the diagnostic radiologist. Abdom Radiol (NY).

[12] Azizzadeh A, Keyhani K, Miller CC 3rd, Coogan SM, Safi HJ, Estrera AL (2009). Blunt traumatic aortic injury: initial experience with endovascular repair. J Vasc Surg.

[13] Lee WA, Matsumura JS, Mitchell RS, Farber MA, Greenberg RK, Azizzadeh A (2011). Endovascular repair of traumatic thoracic aortic injury: clinical practice guidelines of the Society for Vascular Surgery. J Vasc Surg.

[14] Starnes BW, Lundgren RS, Gunn M, Quade S, Hatsukami TS, Tran NT (2012). A new classification scheme for treating blunt aortic injury. J Vasc Surg.

[15] Rabin J, DuBose J, Sliker CW, O’Connor JV, Scalea TM, Griffith BP (2014). Parameters for successful nonoperative management of traumatic aortic injury. J Thorac Cardiovasc Surg.

[16] Kondo Y, Matsui H, Yasunaga H (2019). Characteristics, treatments, and outcomes among patients with abdominal aortic injury in Japan: a nationwide cohort study. World J Emerg Surg.

[17] Sheehan BM, Grigorian A, de Virgilio C, Fujitani RM, Kabutey NK, Lekawa M (2020). Predictors of blunt abdominal aortic injury in trauma patients and mortality analysis. J Vasc Surg.

[18] Moher D, Liberati A, Tetzlaff J, Altman DG, PRISMA Group (2009). Preferred reporting items for systematic reviews and meta-analyses: the PRISMA statement. PLoS Med.

[19] The Joanna Briggs Institute.

[20] Mann HB, Whitney DR (1947). On a test of whether one of two random variables is stochastically larger than the other. Ann Math Stat.

[21] McNutt LA, Wu C, Xue X, Hafner JP (2003). Estimating the relative risk in cohort studies and clinical trials of common outcomes. Am J Epidemiol.

[22] Akaike H (1974). A new look at the statistical model identification. IEEE Trans Autom Control.

[23] Zhang Y (2002). A semiparametric pseudolikelihood estimation method for panel count data. Biometrika.

[24] Warner P (2013). Testing association with Fisher’s exact test. J Fam Plann Reprod Health Care.

[25] Michaels AJ, Gerndt SJ, Taheri PA, Wang SC, Wahl WL, Simeone DM (1996). Blunt force injury of the abdominal aorta. J Trauma.

[26] Martí M, Pinilla I, Baudraxler F, Simón MJ, Garzón G (2006). A case of acute abdominal aortic dissection caused by blunt trauma. Emerg Radiol.

[27] de Jesus-Silva SG, Cardoso RS, de Moraes Silva MA, Maringolo LGF, Rodrigues MM, Miranda FA (2014). A case study involving a blunt abdominal trauma leading to disruption of the aortic bifurcation in an infant. J Vasc Bras.

[28] Perry JR, Escobedo EM, Mann FA (2000). Abdominal aortic injury associated with ‘seat belt syndrome’. Emerg Radiol.

[29] Reaney SM, Parker MS, Mirvis SE, Bundschuh CV, Luebbert PD, Vingan HL (1995). Abdominal aortic injury associated with transverse lumbar spine fracture-imaging findings. Clin Radiol.

[30] Soares G, Ibarra R, Ferral H (2003). Abdominal aortic injury in a child: intravenous digital subtraction angiogram (IVDSA) for the diagnosis of pediatric vascular trauma. Pediatr Radiol.

[31] Pisters PW, Heslin MJ, Riles TS (1993). Abdominal aortic pseudoaneurysm after blunt trauma. J Vasc Surg.

[32] Harkin DW, Kirk G, Clements WD (1999). Abdominal aortic rupture in a child after blunt trauma on a soccer field. Injury.

[33] Ballard DH, Kaskas NM, Hamidian Jahromi A, Skweres J, Youssef AM (2014). Abdominal wall hernia and aortic injury secondary to blunt trauma: case report and review of the literature. Int J Surg Case Rep.

[34] Welborn b JR, Sas JL (1969). Acute abdominal aortic occlusion due to nonpenetrating trauma. Am J Surg.

[35] West CA Jr, Johnson LW, Doucet L, Shah M, Khan I, Heldmann M (2011). Acute aortic occlusion in a child secondary to lap-belt injury treated with thromboendarterectomy and primary repair. J Vasc Surg.

[36] Hewitt RL, Grablowsky OM (1970). Acute traumatic dissecting aneurysm of the abdominal aorta. Ann Surg.

[37] Sugimoto T, Omura A, Kitade T, Takahashi H, Koyama T, Kurisu S (2007). An abdominal aortic rupture due to seatbelt blunt injury: report of a case. Surg Today.

[38] Reisman JD, Morgan AS (1990). Analysis of 46 intra-abdominal aortic injuries from blunt trauma: case reports and literature review. J Trauma.

[39] Blanco FC, Powell DM, Guzzetta PC, Burd RS (2011). Aortic bifurcation rupture after blunt abdominal trauma in a child: a case report. J Pediatr Surg.

[40] Yellinek S, Gimelrich D, Merin O, Reissman P, Arkovitz M (2015). Aortic bifurcation tear following blunt trauma in childhood. J Ped Surg Case Rep.

[41] Bass A, Papa M, Morag B, Adar R (1983). Aortic false aneurysm following blunt trauma of the abdomen. J Trauma.

[42] Swischuk LE, Jadhav SP, Chung DH (2008). Aortic injury with chance fracture in a child. Emerg Radiol.

[43] Bergqvist D, Takolander R (1981). Aortic occlusion following blunt trauma of the abdomen. J Trauma.

[44] Lyons AR (1992). Aortic rupture as a result of a sporting injury. Ann Emerg Med.

[45] Sumpio BE, Gusberg RJ (1987). Aortic thrombosis with paraplegia: an unusual consequence of blunt abdominal trauma. J Vasc Surg.

[46] Patel S, Sheahan CM, Fontenot DD, Sheahan MG (2020). Aortic transection after blunt abdominal trauma in a child. Ann Vasc Surg.

[47] Jongkind V, Linsen MA, Diks J, Vos AW, Klinkert P, Rauwerda JA (2009). Aortoiliac reconstruction for abdominal aortic rupture after blunt trauma. J Trauma.

[48] Yoshioka Y, Morimoto Y, Sugimoto T, Arase H, Araki K (2016). Blunt abdominal aortic injury associated with L2 vertebral fracture. Ann Vasc Surg.

[49] Hamill JK, Ramson DM, Davies-Payne D, Sawyer MC, Evans S (2021). Blunt abdominal aortic injury in children associated with lap belts. ANZ J Surg.

[50] Kao CC, Huang TH, Chen CW, Huang YK (2019). Blunt abdominal aortic injury may accompany bowel transection. Interact Cardiovasc Thorac Surg.

[51] Guetta O, Shaked G, Greenberg G, Sebbag G, Czeiger D (2018). Blunt abdominal aortic injury: a hybrid approach to combined injuries. J Endovasc Resusc Trauma Manag.

[52] Amin A, Alexander JB, O’Malley KF, Doolin E (1993). Blunt abdominal aortic trauma in children: case report. J Trauma.

[53] Frydenberg M, Royle JP, Hoare M (1990). Blunt abdominal aortic trauma. Aust N Z J Surg.

[54] Roth SM, Wheeler JR, Gregory RT, Gayle RG, Parent FN, Demasi R (1997). Blunt injury of the abdominal aorta: a review. J Trauma.

[55] Llorente GJ, Gallego MG, Arnaiz AM (1997). Chronic post-traumatic pseudoaneurysm of the abdominal aorta diagnosed by duplex Doppler ultrasonography. A case report. Acta Radiol.

[56] Crepps JT, Rodriguez A (1989). Combined abdominal aortic and visceral artery injury secondary to blunt trauma. Injury.

[57] Meghoo CA, Gonzalez EA, Tyroch AH, Wohltmann CD (2003). Complete occlusion after blunt injury to the abdominal aorta. J Trauma.

[58] Katsoulis E, Tzioupis C, Sparks I, Giannoudis PV (2006). Compressive blunt trauma of the abdomen and pelvis associated with abdominal aortic rupture. Acta Orthop Belg.

[59] Edwards AT, Williams IM, Griffith GH (1990). Damage to the abdominal aorta following crush injury. Injury.

[60] Raghavendran K, Singh G, Arnoldo B, Flynn WJ (2004). Delayed development of infrarenal abdominal aortic pseudoaneurysm after blunt trauma: a case report and review of the literature. J Trauma.

[61] Diaz JA, Campbell BT, Moursi MM, Boneti C, Kokoska ER, Jackson RJ (2006). Delayed manifestation of abdominal aortic stenosis in a child presenting 10 years after blunt abdominal trauma. J Vasc Surg.

[62] Shindo S, Ogata K, Katahira S, Iyori K, Ishimoto T, Kobayashi M (1997). Delayed manifestation of aortic stenosis after blunt abdominal trauma: report of a case. Surg Today.

[63] Tracy TF, Silen ML, Graham MA (1996). Delayed rupture of the abdominal aorta in a child after a suspected handlebar injury. J Trauma.

[64] Berthet JP, Marty-Ané CH, Veerapen R, Picard E, Mary H, Alric P (2003). Dissection of the abdominal aorta in blunt trauma: endovascular or conventional surgical management?. J Vasc Surg.

[65] Vernhet H, Marty-Ané CH, Lesnik A, Chircop R, Serres-Cousiné O, Picard E (1997). Dissection of the abdominal aorta in blunt trauma: management by percutaneous stent placement. Cardiovasc Intervent Radiol.

[66] Wu Q, Sun S, Xie J, Li T, Li H, Bai X (2021). Early clinical diagnosis and treatment of traumatic aortic injury caused by thoracic and abdominal injuries: a series of four cases with literature review. Biomed Res Int.

[67] Murata T, Ogino H, Isogai N, Kashiwagi H, Shimoyama R, Kawachi J (2021). Endovascular aortic repair for abdominal aortic injury complicated with bowel injury due to blunt abdominal trauma: a case report. Int J Surg Case Rep.

[68] Halkos ME, Nicholas J, Kong LS, Burke JR, Milner R (2006). Endovascular management of blunt abdominal aortic injury. Vascular.

[69] Papazoglou KO, Karkos CD, Kalogirou TE, Giagtzidis IT (2015). Endovascular management of lap belt-related abdominal aortic injury in a 9-year-old child. Ann Vasc Surg.

[70] Huang JT, Heckman JT, Gunduz Y, Ohki T (2009). Endovascular management of stenosis of the infrarenal aorta secondary to blunt abdominal aortic trauma in a multiply injured patient. J Trauma.

[71] Picard E, Marty-Ané CH, Vernhet H, Sessa C, Lesnik A, Senac JP (1998). Endovascular management of traumatic infrarenal abdominal aortic dissection. Ann Vasc Surg.

[72] Tobler WD, Tan TW, Farber A (2012). Endovascular repair of a blunt abdominal aortic injury. Int J Angiol.

[73] Voellinger DC, Saddakni S, Melton SM, Wirthlin DJ, Jordan WD, Whitley D (2001). Endovascular repair of a traumatic infrarenal aortic transection: a case report and review. Vasc Surg.

[74] Stahlfeld KR, Mitchell J, Sherman H (2004). Endovascular repair of blunt abdominal aortic injury: case report. J Trauma.

[75] Teruya TH, Bianchi C, Abou-Zamzam AM, Ballard JL (2005). Endovascular treatment of a blunt traumatic abdominal aortic injury with a commercially available stent graft. Ann Vasc Surg.

[76] Idoguchi K, Yamaguchi M, Okada T, Nomura Y, Sugimura K, Okita Y (2012). Endovascular treatment of blunt traumatic abdominal aortic occlusion with kissing stent placement. Cardiovasc Intervent Radiol.

[77] Al Zahrani HA (1991). False aneurysm of the abdominal aorta after blunt trauma. Eur J Vasc Surg.

[78] Sethi GK, Scott SM, Takaro T (1975). False aneurysm of the abdominal aorta due to blunt trauma. Ann Surg.

[79] Almeida AA, Frydman GM, Walker PJ (1997). Hyperextension injury causing abdominal aortic rupture. Eur J Vasc Endovasc Surg.

[80] Kalina M, Donovan M, Giberson F, Tinkoff G (2010). Inframesocolic abdominal aortic injury and lumbar vertebral body fracture secondary to hyperextension with blunt trauma. Eur J Trauma Emerg Surg.

[81] Coimbra R, Yang J, Hoyt DB (1996). Injuries of the abdominal aorta and inferior vena cava in association with thoracolumbar fractures: a lethal combination. J Trauma.

[82] Marty-Ané CH, Alric P, Prudhomme M, Chircop R, Serres-Cousiné O, Mary H (1996). Intravascular stenting of traumatic abdominal aortic dissection. J Vasc Surg.

[83] Raguan B, Shnaker A, Fajer S (2018). Isolated abdominal injury of blunt aortic trauma: two case reports and review of the literature. J Endovasc Resusc Trauma Manag.

[84] Khattak MN, Olivero EV, Curi MA, Dhadwal AK, Padberg FT, Huang JT (2015). Isolated blunt abdominal aortic injury without concomitant abdominal injuries treated with endovascular stent grafting. J Vasc Surg Cases.

[85] Dudko S, Kusz D, Ziaja D, Kusz B (2012). Laceration of abdominal aorta by a fragment of fractured L2 vertebral body after a low-energy injury: a case report and review of literature. Spine.

[86] Khanna PC, Rothenbach P, Guzzetta PC, Bulas DI (2007). Lap-belt syndrome: management of aortic intimal dissection in a 7-year-old child with a constellation of injuries. Pediatr Radiol.

[87] Vuorisalo S, Railo M, Lappalainen K, Aho P, Lepäntalo M (2005). Low-energy blunt abdominal aortic trauma in an underweighted man. Eur J Vasc Endovasc Surg.

[88] Diez AA, Monteiro EM, Hoyos YG, Legrand JL (2018). Management and evolution of a wallstent migration in a pediatric blunt traumatic abdominal aortic injury. Ann Vasc Surg.

[89] Parrish DW, Barnhorst A, Trebska-McGowan K, Amendola M, Haynes JH (2015). Nonoperative management of pediatric aortic injury with seat belt syndrome. Ann Vasc Surg.

[90] Macbeth A, Malone JM, Norton LW, Peltier LF (1982). Paralysis and aortic thrombosis following blunt abdominal trauma. J Trauma.

[91] Nishimura K, Kanaoka Y, Ikebuchi M, Hiroe T, Tachibana M, Ishiguro S (2000). Percutaneous balloon fenestration in a case of traumatic abdominal aortic dissection with lower extremity ischemia. J Vasc Surg.

[92] Balas P, Stamatopoulos C, Chagitheophilou C (1974). Post-traumatic aneurysm of the abdominal aorta. Angiology.

[93] Freni L, Barbetta I, Mazzaccaro D, Settembrini AM, Dallatana R, Tassinari L (2013). Seat belt injuries of the abdominal aorta in adults–case report and literature review. Vasc Endovascular Surg.

[94] Shweiki E, Klena JW, Halm KA, Leonard DJ (2000). Seat belt injury to the abdominal aorta. Am J Emerg Med.

[95] Tomic I, Dragas M, Vasin D, Loncar Z, Fatic N, Davidovic L (2018). Seat-belt abdominal aortic injury-treatment modalities. Ann Vasc Surg.

[96] Clyne CA, Ashbrooke EA (1985). Seat-belt aorta: isolated abdominal aortic injury following blunt trauma. Br J Surg.

[97] Warrian RK, Shoenut JP, Iannicello CM, Sharma GP, Trenholm BG (1988). Seatbelt injury to the abdominal aorta. J Trauma.

[98] Campbell DK, Austin RF (1969). Seat-belt injury: injury of the abdominal aorta. Radiology.

[99] David D, Blumenberg RM (1970). Subintimal aortic dissection with occlusion after blunt abdominal trauma. Arch Surg.

[100] Crabb GM, McQuillen KK (2006). Subtle abdominal aortic injury after blunt chest trauma. J Emerg Med.

[101] Huh U, Lee CW, Kim SH, Park CI, Chung SW, Song S (2018). Successful treatment using a kissing stent for blunt abdominal aortic injury: a case report. Cardiovasc Intervent Radiol.

[102] Nicholls S, Karmy-Jones R (2011). Ten-year follow-up after endovascular repair of traumatic abdominal aortic rupture. Innovations (Phila).

[103] Qureshi A, Roberts N, Nicholson A, Johnson B (1997). Three-dimensional reconstruction by spiral computed tomography to locate aortic tear following blunt abdominal trauma. Eur J Vasc Endovasc Surg.

[104] Borja AR, Lansing AM (1970). Thrombosis of the abdominal aorta caused by blunt trauma. J Trauma.

[105] Heck JM, Bittles MA (2009). Traumatic abdominal aortic dissection in a 16-month-old child. Pediatr Radiol.

[106] Yulevich A, Singer-Jordan J, Grozovsky Y, Zonis Z, Sweed Y (2007). Traumatic abdominal aortic dissection in a child. J Trauma.

[107] Gunn M, Campbell M, Hoffer EK (2007). Traumatic abdominal aortic injury treated by endovascular stent placement. Emerg Radiol.

[108] Thal ER, Perry MO, Crighton J (1971). Traumatic abdominal aortic occlusion. South Med J.

[109] Andrey V, Bettschart V, Ducrey N, Constantin C, Genin B (2013). Traumatic abdominal aortic rupture treated by endovascular stent placement in an 11-year-old boy. J Pediatr Surg Case Rep.

[110] Matolo NM, Danto LA, Wolfman EF (1974). Traumatic aneurysm of the abdominal aorta. Report of two cases and review of the literature. Arch Surg.

[111] Boumzebra D, Ettaoumi Y, Yaacoubi M, Haddani J, Fehri M, Mehadji BA (2005). Traumatic aneurysm of the bifurcation of the abdominal aorta in an infant. A case report. Eur J Pediatr Surg.

[112] Anomah Ngu V, Konstam PG (1965). Traumatic dissecting aneurysm of the abdominal aorta. Br J Surg.

[113] Desai SC, Chute DJ, Desai BC, Koloski ER (2008). Traumatic dissection and rupture of the abdominal aorta as a complication of the Heimlich maneuver. J Vasc Surg.

[114] Gordon CR, Eakins JS, DeAngelo FJ, Ross SE, Ierardi RP (2007). Traumatic fall injury causing a displaced vertebral body fracture with concomitant abdominal aortic injury. J Trauma.

[115] Brunsting LA, Ouriel K (1988). Traumatic fracture of the abdominal aorta. Rupture of a calcified abdominal aorta with minimal trauma. J Vasc Surg.

[116] Addicott B, Schmitz K (2014). Traumatic handle bar injury with aortic disruption. Pediatr Radiol.

[117] Godry H, Rölleke G, Mumme A, Schildhauer TA, Gothner M (2014). Traumatic infra-renal aortic dissection after a high-energy trauma: a case report of a primary missed diagnosis. Orthop Rev.

[118] Coz S, Merson L, Morel N, Dabadie P (2010). Traumatic rupture of the abdominal aorta after spinal fracture from low fall. Ann Fr Anesth Reanim.

[119] Walker AG, Walker RM (1961). Traumatic thrombosis of the aorta. Br Med J.

[120] Chiarini V, Coniglio C, Ferri E, Lupi C, Tugnoli G, Montanari N (2019). Use of REBOA as a bridge to endovascular aortic repair in blunt abdominal aortic injury. J Endovasc Resusc Trauma Manag.

[121] Abed H, Ball WR, Stone T, Houghton A (2017). Very late rupture of a post-traumatic abdominal aortic pseudoaneurysm. BMJ Case Rep.

[122] Lock JS, Huffman AD, Johnson RC (1987). Blunt trauma to the abdominal aorta. J Trauma.

[123] Gilani R, Saucedo-Crespo H, Scott BG, Tsai PI, Wall MJ, Mattox KL (2012). Endovascular therapy for overcoming challenges presented with blunt abdominal aortic injury. Vasc Endovasc Surg.

[124] Jovanovic M, Radojkovic M, Djordjevic P, Rancic D, Jovanovic N, Rancic Z (2017). Recycling and reinforcing intimomedial flap of the infrarenal aorta using anterior longitudinal ligament in patients with acute trauma with bowel injuries. Vasc Endovasc Surg.

[125] Feliciano DV (1988). Abdominal vascular injuries. Surg Clin North Am.

[126] Szilagyi DE, Hageman JH, Smith RF, Elliott JP (1978). Spinal cord damage in surgery of the abdominal aorta. Surgery.

[127] Mellnick VM, McDowell C, Lubner M, Bhalla S, Menias CO (2012). CT features of blunt abdominal aortic injury. Emerg Radiol.

[128] Inaba K, Kirkpatrick AW, Finkelstein J, Murphy J, Brenneman FD, Boulanger BR (2001). Blunt abdominal aortic trauma in association with thoracolumbar spine fractures. Injury.

[129] Harris DG, Drucker CB, Brenner ML, Sarkar R, Narayan M, Crawford RS (2013). Patterns and management of blunt abdominal aortic injury. Ann Vasc Surg.

[130] McNally MM, Univers J (2018). Acute limb ischemia. Surg Clin North Am.

[131] Hess CN, Huang Z, Patel MR, Baumgartner I, Berger JS, Blomster JI (2019). Acute limb ischemia in peripheral artery disease. Circulation.

